# Progressive cerebral infarction as the presenting feature of ANCA-associated vasculitis with concurrent Evans syndrome: a case report and literature review

**DOI:** 10.3389/fimmu.2026.1764226

**Published:** 2026-05-20

**Authors:** HanYe Yuan, Tianhao Zhang, Minghui Du, Zhigang Liang

**Affiliations:** 1 Department of Neurology, Yuhuangding Hospital Affiliated to Qingdao University, Yantai, China; 2 Shandong Provincial Key Laboratory of Neuroimmune Interaction and Regulation, Yantai, China; 3 National Clinical Medical Research Center for Neurological Diseases Regional Subcenter, Yantai, China

**Keywords:** ANCA-associated vasculitis, Evans syndrome, immunosuppression, ischemic stroke, plasma exchange

## Abstract

Antineutrophil cytoplasmic antibody (ANCA)-associated vasculitis (AAV) is a systemic autoimmune disease, and central nervous system involvement, though uncommon, can be a severe manifestation. The co-occurrence of AAV and Evans syndrome (ES) is exceptionally rare and may reflect broader immune dysregulation. A 72-year-old female presented with progressive right-sided hemiplegia and mixed aphasia. Imaging revealed multiple acute cerebral infarcts. Laboratory findings showed anemia, thrombocytopenia, acute kidney injury, and elevated inflammatory markers. Serology confirmed myeloperoxidase (MPO)-ANCA positivity, supporting a diagnosis of AAV, most consistent with microscopic polyangiitis. Subsequent hematological workup supported concurrent autoimmune cytopenias consistent with probable ES. Despite corticosteroids and intravenous immunoglobulin, her condition deteriorated with left middle cerebral artery occlusion; during hospitalization, this deterioration coincided with a SARS-CoV-2 infection. Management was escalated to therapeutic plasma exchange and cyclophosphamide. This regimen stabilized renal and hematological parameters, although severe neurological deficits and thrombocytopenia persisted. This case highlights that stroke may be a catastrophic presenting feature of AAV and that concurrent autoimmune cytopenias may add substantial diagnostic and therapeutic complexity. Early ANCA testing in cryptogenic or progressive stroke with systemic involvement is vital, and management requires careful balance between immunosuppression and infection risk.

## Introduction

Antineutrophil cytoplasmic antibody (ANCA)-associated vasculitis (AAV) is a heterogeneous group of autoimmune diseases characterized by necrotizing inflammation primarily affecting small vessels. Its clinical spectrum includes granulomatosis with polyangiitis (GPA), microscopic polyangiitis (MPA), and eosinophilic granulomatosis with polyangiitis (EGPA) (1). Although central nervous system involvement is uncommon, patients with AAV exhibit a significantly elevated risk of acute cerebral infarction. Diagnosis may be particularly challenging when stroke is the initial or primary presentation, as it often mimics atherosclerotic cerebrovascular disease (2, 3). Evans syndrome (ES) is a rare autoimmune disorder characterized by the presence of at least two autoimmune cytopenias (AICs), including immune thrombocytopenia (ITP), autoimmune hemolytic anemia (AIHA), and autoimmune neutropenia (AIN) (4). The association between ES and AAV is extremely rare (5, 6). The coexistence of these two entities may reflect broader immune dysregulation and poses significant therapeutic challenges (6–8). We report a case of progressive cerebral infarction in a patient subsequently diagnosed with AAV concurrent with ES. This case highlights the diagnostic challenges of AAV with predominant central nervous system (CNS) involvement and underscores the complex management difficulties associated with its co-occurrence with ES.

## Case report

A 72-year-old female was transferred to our neurology ward with progressive right-sided hemiplegia and mixed aphasia over one month. One month prior, she had experienced acute right-sided limb weakness. Cranial magnetic resonance imaging (MRI) had shown acute cerebral infarction in the splenium of the corpus callosum and left frontoparietal region, for which antiplatelet therapy was initiated. Her medical history was significant for coronary artery disease (status post stenting), atrial septal defect (status post repair), and a remote history of self-reported “nephritis”.

**Figure 1:**
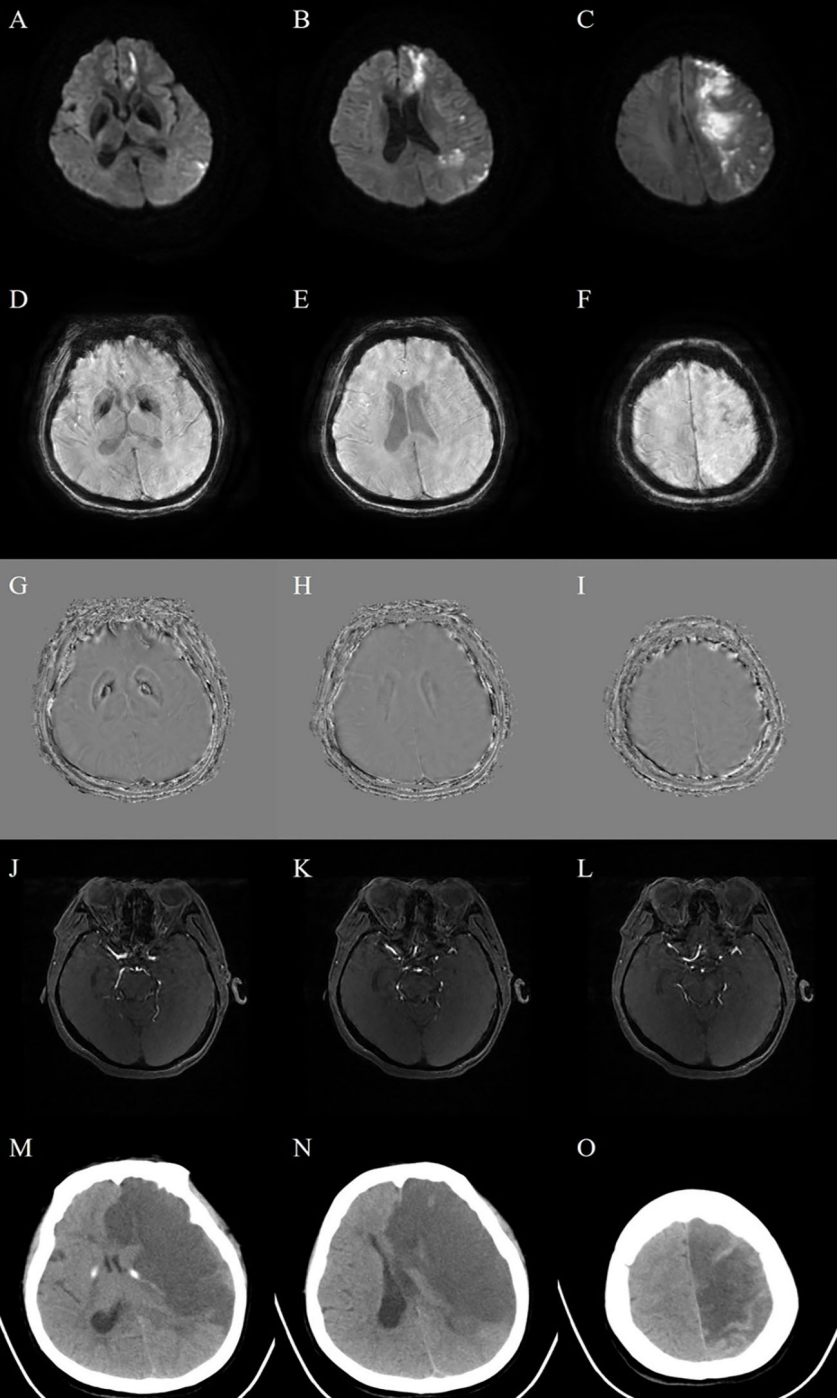
Neuroimaging findings at admission and during clinical deterioration. **(A–C)** Admission diffusion-weighted imaging (DWI): Hyperintense signals are evident in the left frontoparietal cortex and deep gray nuclei, consistent with acute ischemic infarcts involving multiple vascular territories. **(D–I)** Susceptibility-weighted imaging (SWI): Focal susceptibility blooming artifacts within the infarcted regions indicate hemorrhagic transformation with microhemorrhages. **(J–L)** Axial MRA source images demonstrated ≥50% luminal narrowing in the M1 segment of the left middle cerebral artery. **(M–O)** Emergency cranial computed tomography (CT) on day 16: Worsening hypoattenuation in the left hemisphere reflects progressive infarction, accompanied by effacement of the left lateral ventricle and subfalcine herniation.

On admission, the patient was alert. Vital signs were as follows: temperature 36.6 °C, pulse 88/min, respiratory rate 18/min, and blood pressure 160/90 mmHg. General physical examination revealed an anemic appearance and palpable purpura on both lower extremities. Neurological examination revealed incomplete mixed aphasia, right-sided hemiplegia (muscle strength 0/5 in upper limb, 1/5 in lower limb). Reflex testing showed a positive Babinski sign on the right. The National Institutes of Health Stroke Scale (NIHSS) score was 14 points. Cranial MRI revealed new acute left frontoparietal cortical and multifocal left hemispheric infarcts (**Figures 1A–C**). Susceptibility-weighted imaging (SWI) showed microhemorrhages within these infarcts (**Figures 1D–I**). Formal cranial magnetic resonance angiography (MRA) was suboptimal because of poor cooperation. However, axial MRA source images demonstrated ≥50% luminal narrowing in the M1 segment of the left middle cerebral artery (**Figures 1J–L**). Chest computed tomography (CT) demonstrated bilateral diffuse ground-glass opacities, suggestive of inflammation. An electrocardiogram showed sinus rhythm, and echocardiography revealed a left ventricular ejection fraction of 64% with no residual interatrial shunt after atrial septal defect closure. Carotid ultrasound demonstrated intima-media thickening with plaque formation bilaterally, and lower-extremity venous ultrasound showed no evidence of deep venous thrombosis. Renal ultrasound demonstrated increased parenchymal echogenicity. Holter monitoring or extended cardiac rhythm monitoring was not performed during hospitalization.

Laboratory investigations showed hemoglobin 79 g/L (reference range: 115–150 g/L), platelets 109 × 10^9^/L (reference range: 125–350 × 10^9^/L), creatinine 187 μmol/L (reference range: 41–81 μmol/L), and C-reactive protein 85.60 mg/L (reference range: 0.00–6.00 mg/L). Baseline hemolysis-related markers showed lactate dehydrogenase (LDH) of 218 U/L and indirect bilirubin of 8.1 μmol/L. Urinalysis showed hematuria (278 red blood cells per high-power field) and proteinuria (+). Over the next several days, her cytopenias worsened (hemoglobin 58 g/L, platelets 68 × 10^9^/L), prompting packed red blood cell transfusion. On day 7, serological testing confirmed a positive myeloperoxidase (MPO)-ANCA titer (>200 RU/mL) with negative proteinase 3 (PR3)-ANCA, supporting a diagnosis of ANCA-associated vasculitis (AAV), most consistent with microscopic polyangiitis (MPA) (9). Antiplatelet therapy was discontinued, and intravenous methylprednisolone (40 mg daily) was initiated. Because of progressive anemia and thrombocytopenia, concurrent AICs were suspected in the framework of Evans syndrome (ES) (4). Intravenous immunoglobulin (IVIG) was administered over five days starting on day 12.

On day 16, her condition deteriorated acutely, with fever and somnolence. Emergency cranial CT showed worsening left hemispheric infarction with evidence of subfalcine herniation and occlusion of the left middle cerebral artery (**Figures 1M–O**). Laboratory investigations showed hemoglobin 66 g/L and platelets 49 × 10^9^/L. Additional hematological evaluation was performed at this stage. The direct antiglobulin test was positive for anti-IgG and C3, and the reticulocyte percentage was 2.98%. Hemolysis-related markers showed LDH of 316 U/L, indirect bilirubin of 10.5 μmol/L, and haptoglobin of 3.77 g/L. Although haptoglobin was not decreased and indirect bilirubin remained within the reference range, the combination of progressive anemia, thrombocytopenia, positive direct antiglobulin test, and reticulocytosis supported concurrent AICs consistent with probable Evans syndrome. Eltrombopag was added. Concurrently, she tested positive for SARS-CoV-2 and received antiviral therapy with molnupiravir. Serial chest CT during hospitalization showed dynamic inflammatory changes, with overall improvement on interim follow-up compared with the admission CT, but slight progression of the right upper lobe lesion on later follow-up imaging. Because of refractory disease activity, two sessions of therapeutic plasma exchange were performed on hospital day 30, and cyclophosphamide was added to the treatment regimen.

After treatment escalation, follow-up hemolysis-related testing showed that LDH decreased to 252 U/L and indirect bilirubin was 13.2 μmol/L. Thereafter, her renal function and anemia improved significantly (discharge hemoglobin 87 g/L, creatinine 105 μmol/L), although thrombocytopenia persisted (platelet count 48 × 10^9^/L). Follow-up cranial CT demonstrated a reduction in the mass effect associated with the cerebral infarction. She was discharged on day 39 with severe residual neurological deficits (NIHSS score 13). Discharge medications included cyclophosphamide and a tapering dose of prednisone for ongoing immunosuppressive therapy. Unfortunately, the patient’s overall condition remained fragile. She passed away two months after discharge. The clinical timeline, key findings, and management are summarized in **Table 1**.

**Table 1:** Clinical timeline, key findings, and management of the presented case.

Timeline	Clinical manifestations and investigations	Management and treatment
First Event (1 month prior)	• Acute right-sided limb weakness. • Cranial MRI showed acute cerebral infarction involving the splenium of the corpus callosum and the left frontoparietal region.	• Antiplatelet therapy initiated.
Day 1 (Admission)	• Neurological deficits: right-sided hemiplegia, aphasia, NIHSS score 14. • Cranial MRI showed new multifocal left hemispheric infarcts with microhemorrhages. • Laboratory: Anemia, thrombocytopenia, acute kidney injury, elevated CRP; baseline LDH 218 U/L and indirect bilirubin 8.1 μmol/L.	• Transferred to neurology ward.
Day 7	• MPO-ANCA strongly positive (>200 RU/mL).	• Diagnosis of AAV established. • Glucocorticoid therapy initiated.
Day 12	• Worsening anemia and thrombocytopenia.	• Intravenous immunoglobulin therapy initiated (5-day course).
Day 16	• Acute deterioration with fever and somnolence. • Cranial CT showed worsening left hemispheric infarction with subfalcine herniation and left MCA occlusion. • SARS-CoV-2 test positive. • Direct antiglobulin test (anti-IgG and C3) positive; reticulocyte percentage 2.98%; LDH 316 U/L, indirect bilirubin 10.5 μmol/L, and haptoglobin 3.77 g/L.	• Probable Evans syndrome/concurrent AICs considered. • Eltrombopag added; antiviral therapy initiated after SARS-CoV-2 positivity.
Day 30	• Refractory cytopenias, including anemia and thrombocytopenia.	• Therapeutic plasma exchange performed. • Cyclophosphamide added.
Day 36-39	• Improvement in hemoglobin and renal function. • Persistent thrombocytopenia. • Severe neurological deficits (NIHSS score 13). • Follow-up cranial CT showed reduced mass effect. • Follow-up LDH 252 U/L and indirect bilirubin 13.2 μmol/L.	• Discharged on cyclophosphamide and prednisone.
2 months after discharge	• Patient died	

MRI, cranial magnetic resonance imaging; NIHSS, National Institutes of Health Stroke Scale; CRP, C-reactive protein; LDH, lactate dehydrogenase; MPO, myeloperoxidase; ANCA, antineutrophil cytoplasmic antibody; AAV, Antineutrophil Cytoplasmic Antibody-Associated Vasculitis; MCA, top cerebral artery; AICs, autoimmune cytopenias; CT, computed tomography.

## Discussion

**Figure 2:**
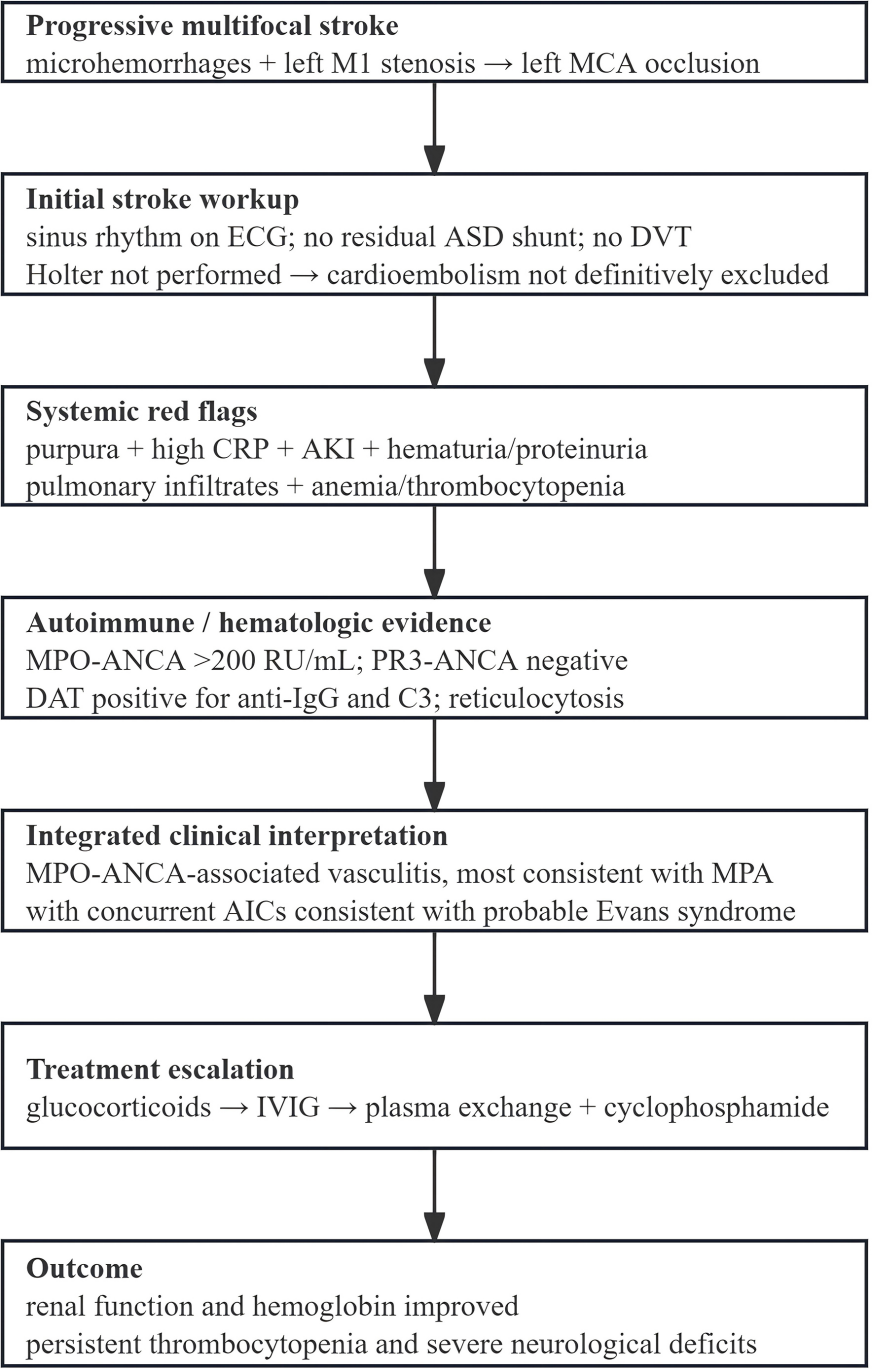
Clinical reasoning pathway. The pathway illustrates how progressive multifocal stroke, incomplete exclusion of cardioembolism, systemic vasculitic red flags, myeloperoxidase-antineutrophil cytoplasmic antibody (MPO-ANCA) positivity, and autoimmune cytopenic features led to the integrated diagnosis of antineutrophil cytoplasmic antibody-associated vasculitis (AAV) with concurrent autoimmune cytopenias (AICs) consistent with probable Evans syndrome and subsequent treatment escalation.

This case report describes an elderly female patient presenting with progressive cerebral infarction and ANCA-associated vasculitis, ultimately diagnosed with microscopic polyangiitis complicated by concurrent AICs consistent with probable Evans syndrome. Her condition later deteriorated during hospitalization, coinciding with SARS-CoV-2 infection, progressing to left middle cerebral artery occlusion and extensive cerebral infarction. The complexity of this case and its multisystem involvement underscore the diagnostic and therapeutic challenges of AAV with ES, particularly the treatment dilemmas and poor prognosis associated with concurrent infection. The clinical reasoning pathway for the diagnosis and treatment escalation is summarized in **Figure 2**.

There is a higher incidence of stroke in AAV patients compared with the general population (3, 10). Unlike typical atherosclerotic or cardioembolic stroke, AAV-related cerebrovascular involvement may affect multiple vascular territories and involve both small and, less commonly, large vessels (2, 11). Although AAV is classically categorized as a small-vessel vasculitis, recent studies have increasingly recognized large-vessel involvement as a rare but genuine vascular phenotype (12, 13). This distinction stems from the nature of vasculitis, which can induce inflammation and damage in vessels of various sizes throughout the body, including the brain (2, 7). Previously reported AAV-related stroke cases have more often presented with recurrent or multifocal infarction, sometimes accompanied by hemorrhagic or microhemorrhagic change (11, 14–16) Selected reported cases of AAV with stroke, with or without Evans syndrome, are summarized in **Table 2**. Treatment in reported cases has commonly included glucocorticoids together with cyclophosphamide, while rituximab or plasma exchange may be considered in selected severe presentations (9, 16–18). By contrast, the coexistence of AAV and Evans syndrome appears to be exceedingly uncommon. The limited literature on relevant overlap cases suggests that presentations are more often dominated by hematologic or other systemic manifestations than by catastrophic progressive stroke (19, 20). In this respect, our patient may expand the reported clinical spectrum of this overlap syndrome.

**Table 2:** Selected reported cases of AAV with stroke ± Evans syndrome.

Author	AAV subtype	Stroke type	Treatment	Outcome
Wu et al., 2024 (28)	MPO/p-ANCA-positive AAV, highly suggestive of MPA	Repeated small infarcts with pontine microhemorrhage and corpus callosum “snowball-like” lesion	Glucocorticoids, tocilizumab for induction, and mycophenolate mofetil for maintenance	Multidisciplinary individualized treatment achieved good clinical effects.
Uppal et al., 2020 (29)	C-ANCA-positive AAV; not clearly classifiable as GPA or EGPA	Acute/subacute bilateral occipito-parieto-temporal and thalamic infarcts with hemorrhagic transformation	Pulse methylprednisolone followed by cyclophosphamide, oral prednisolone, and rehabilitation	Consciousness and limb strength partially improved; delayed presentation was associated with incomplete motor recovery.
Esfahani et al., 2017 (30)	PR3/c-ANCA-positive AAV	Acute ischemic stroke followed by multiple bilateral intracerebral hemorrhages after IV tPA	IV tPA before AAV was recognized; subsequently corticosteroids and rituximab	Marked neurological and renal improvement at 3-month follow-up; modified Rankin Scale score was 3.
Wakisaka et al., 2014 (14)	CNS-limited MPO-ANCA-associated vasculitis	Recurrent multifocal ischemic strokes involving bilateral cerebral regions despite antithrombotic therapy	Methylprednisolone pulse therapy followed by oral prednisolone	Inflammatory response improved; no further stroke recurrence before rehabilitation transfer.
Isoda et al., 2010 (15)	MPO-ANCA-positive MPA	Right corona radiata infarction followed by right thalamic hemorrhage	Prednisolone, intravenous cyclophosphamide, methylprednisolone pulse therapy, plasma exchange, and double-filtration plasmapheresis	MPA activity and renal function improved, but neurological deficits did not substantially change; patient was discharged.
Ghinoi et al., 2010 (16)	PR3/c-ANCA-positive AAV; clinical phenotype not fully classifiable, MPA considered possible	Multiple left hemispheric ischemic lesions with distal left MCA branch stenoses; later microhemorrhagic lesions	Glucocorticoids, cyclophosphamide, and intravenous immunoglobulin; plasma exchange was also used earlier in the disease course	Clinical status and MRI/MRA findings improved; no recurrence of brain vasculitis during follow-up.

MPO, myeloperoxidase; p-ANCA, perinuclear antineutrophil cytoplasmic antibody; AAV, antineutrophil cytoplasmic antibody-associated vasculitis; MPA, microscopic polyangiitis; C-ANCA/c-ANCA, cytoplasmic antineutrophil cytoplasmic antibody; GPA, granulomatosis with polyangiitis; EGPA, eosinophilic granulomatosis with polyangiitis; PR3, proteinase 3; IV, intravenous; tPA, tissue plasminogen activator; CNS, central nervous system; MCA, middle cerebral artery; MRI, magnetic resonance imaging; MRA, magnetic resonance angiography.

From a practical perspective, several red flags should prompt consideration of vasculitis in stroke patients, particularly when the stroke is progressive, recurrent, multifocal, or accompanied by hemorrhagic transformation or microhemorrhages. Additional clues include arterial stenosis or occlusion that progresses despite standard antithrombotic therapy, markedly elevated inflammatory markers, fever or constitutional symptoms, palpable purpura or other vasculitic skin lesions, renal abnormalities such as acute kidney injury, hematuria or proteinuria, pulmonary infiltrates or alveolar hemorrhage, chronic sinonasal or otologic symptoms, peripheral neuropathy or mononeuritis multiplex, and unexplained cytopenias. When these features coexist, early evaluation with inflammatory markers, urinalysis, renal function testing, chest imaging, autoimmune screening, and ANCA testing should be considered, especially in cryptogenic or atypical stroke presentations (2, 11, 16, 21).

In our patient, several features supported attributing the stroke primarily to active AAV rather than to conventional cerebrovascular disease alone: multifocal infarction with microhemorrhagic change, progressive M1 stenosis evolving to occlusion despite antiplatelet therapy, marked systemic inflammation, MPO-ANCA positivity, renal abnormalities, purpura, and autoimmune cytopenias (2, 11, 12, 16). At the same time, alternative explanations must be acknowledged. Atherosclerotic disease was plausible given her age, prior coronary artery disease, carotid plaque, and later intracranial arterial calcification. Cardioembolism could not be definitively excluded because Holter monitoring, repeated rhythm monitoring, or extended outpatient rhythm monitoring was not performed. This limitation is clinically relevant because short ECG recordings may miss paroxysmal atrial fibrillation, and current stroke secondary-prevention guidance emphasizes rhythm monitoring for occult atrial fibrillation when no other cause of stroke is identified (22, 23). Nevertheless, the available sinus rhythm on electrocardiogram, absence of residual shunt after atrial septal defect closure, negative venous ultrasound, and prominent systemic vasculitic features made cardioembolism less compelling. Thus, the most balanced interpretation is that the stroke was primarily related to active AAV, while concomitant atherosclerotic disease, cardioembolism, and other conventional contributors could not be fully excluded.

The occurrence of probable ES added a layer of complexity to the clinical presentation. ES is characterized by the concurrent or sequential destruction of red blood cells and platelets mediated by autoantibodies (5). Notably, 21% to 50% of ES cases are secondary, often associated with autoimmune diseases, immunodeficiencies, and lymphoproliferative disorders (4). However, the rare coexistence of AAV and ES may reflect a broader breakdown of immune tolerance rather than two entirely unrelated autoimmune processes (7, 8). In AAV, loss of immune tolerance permits autoreactive B-cell responses and ANCA generation, while pathogenic ANCA promotes neutrophil activation, endothelial injury, and complement amplification (7). In ES, immune dysregulation leads to erythrocyte- and platelet-directed autoantibodies, resulting in autoimmune hemolysis and thrombocytopenia; complement-mediated injury is particularly relevant to the hemolytic component (6, 7, 24). Although this shared immunopathogenic framework cannot be proven definitively in a single case, it helps explain the patient’s concurrent vasculitic organ injury and severe cytopenias.

Following the confirmation of MPO-ANCA positivity, glucocorticoid therapy was initiated promptly because the patient was considered to have organ- or life-threatening AAV, given her progressive CNS ischemic involvement and renal abnormalities (2, 9). Because cytopenias worsened during hospitalization, IVIG was added in the setting of suspected concurrent AICs. After further neurological and hematologic deterioration, the coexistence of progressive anemia, thrombocytopenia, a positive direct antiglobulin test, and reticulocytosis supported concurrent AICs consistent with probable Evans syndrome. However, biochemical evidence of hemolysis was incomplete because haptoglobin was not decreased and indirect bilirubin remained within the reference range. Therefore, ES was considered a probable clinical diagnosis rather than a diagnosis fully confirmed by concordant hemolysis markers. Therapeutic plasma exchange was subsequently used as rescue therapy for fulminant refractory disease, reflecting the need for rapid control of ongoing disease activity, and cyclophosphamide was then initiated as remission-induction therapy for severe AAV (2, 9, 15, 18). The subsequent improvement in renal function and hemoglobin suggested a partial hematologic and renal response to treatment escalation, although thrombocytopenia and severe neurological deficits persisted. This case underscores the importance of infection prevention and close monitoring for infectious, hematologic, and renal complications during intensive immunosuppressive therapy (9, 18). SARS-CoV-2 infection, detected during hospitalization, was unlikely to account for the initial stroke presentation; however, it may have acted as an aggravating factor by amplifying inflammation, coagulation activation, and overall management complexity (25, 26).

Despite treatment escalation, the patient was left with severe neurological deficits. This underscores the potentially catastrophic and often irreversible nature of CNS damage associated with active vasculitic cerebrovascular involvement (11, 27). This case highlights that systemic vasculitis should be considered in the differential diagnosis of cryptogenic or progressive stroke, particularly when accompanied by systemic symptoms and multiorgan laboratory abnormalities (2, 11). Early ANCA serological testing is crucial for timely diagnosis and may facilitate prompt initiation of appropriate immunosuppressive treatment (21).

This report has several limitations. First, renal biopsy was not performed; therefore, AAV-related glomerulonephritis could not be histologically confirmed. Second, bronchoscopy was not performed, so pulmonary vasculitic involvement or alveolar hemorrhage could not be established with certainty despite serial chest CT follow-up. Third, Holter monitoring, repeated rhythm monitoring, or extended outpatient rhythm monitoring was unavailable; consequently, occult paroxysmal atrial fibrillation and a cardioembolic contribution could not be definitively excluded. Finally, biochemical evidence of hemolysis was incomplete, as haptoglobin was not decreased and indirect bilirubin remained within the reference range.

## Conclusion

This case highlights several important clinical lessons. First, acute or progressive cerebral infarction, particularly when multifocal and accompanied by systemic inflammatory features, can be the presenting feature of ANCA-associated vasculitis. Second, the rare concurrence of AAV with AICs consistent with probable Evans syndrome may reflect broader immune dysregulation and may contribute to severe hematologic and systemic manifestations. Third, the clinical course illustrates the challenge of balancing immunosuppression against the risk of intercurrent infection, as evidenced by the patient’s deterioration during hospitalization with SARS-CoV-2 infection. Despite escalated therapy with plasma exchange and cyclophosphamide, the patient sustained severe neurological deficits. This outcome emphasizes the critical importance of early diagnosis. Therefore, this case supports considering early ANCA testing in selected patients with cryptogenic or progressive stroke accompanied by systemic inflammatory or multiorgan features.

## Data Availability

The raw data supporting the conclusions of this article will be made available by the authors, without undue reservation.
